# Supplementary material for: FoxO suppresses endoplasmic reticulum stress to inhibit growth of Tsc1-deficient tissues under nutrient restriction
Source: eLife. 2020 Jun 11;9:e53159. doi: 10.7554/eLife.53159 (PMC7289595; doi:10.7554/eLife.53159)
Supplement: Supplementary file 1. [file elife-53159-supp1.docx]

**Supplementary File 1: Key Resources Table**

| **Key Resources Table** | | | | |
| --- | --- | --- | --- | --- |
| **Reagent type (species) or resource** | **Designation** | **Source or reference** | **Identifiers** | **Additional information** |
| Genetic reagent (*Drosophila melanogaster*) | *hsFLP* | PMID: 8440019 | FLYB:FBtp0001101 |  |
| Genetic reagent (*D. melanogaster*) | *eyFLP Act>CD2>Gal4* | PMID: 23874212 | N/A |  |
| Genetic reagent (*D. melanogaster*) | *eyFlp gl-LacZ* | PMID:  10648243 | N/A |  |
| Genetic reagent (*D. melanogaster*) | *Act>CD2>Gal4 UAS-RFP* | Bloomington Drosophila Stock Center (BDSC) | BDSC:30558; RRID:BDSC_30558 |  |
| Genetic reagent (*D. melanogaster*) | wt; ctrl | PMID: 8404527 | N/A | *FRT82 iso* |
| Genetic reagent (*D. melanogaster*) | *FRT82 foxo^∆94^* | PMID: 21443682 | FLYB:FBal0269838 |  |
| Genetic reagent (*D. melanogaster*) | *FRT82 Tsc1^Q87X^* | PMID: 11348591 | FLYB:FBal0123965 |  |
| Genetic reagent (*D. melanogaster*) | *FRT82 Tsc1^Q87X^ foxo^∆94^* | PMID: 29677182 | N/A |  |
| Genetic reagent (*D. melanogaster*) | *FRT82 cl w^+^* | PMID:  10648243 | N/A |  |
| Genetic reagent (*D. melanogaster*) | *tub-Gal4 FRT82 tub-Gal80 ubi-GFP* | This paper | N/A | See Figure 1 |
| Genetic reagent (*D. melanogaster*) | *tub-Gal80^ts^* | BDSC | BDSC:7108; RRID:BDSC_7108 |  |
| Genetic reagent (*D. melanogaster*) | ctrl | Vienna Drosophila Resource Center (VDRC) | VDRC:47097 | RNAi of *CG1315* |
| Genetic reagent (*D. melanogaster*) | *foxo; foxo^Ri^* | VDRC | VDRC:107786 | RNAi of *foxo* |
| Genetic reagent (*D. melanogaster*) | *Tsc1; Tsc1^Ri^* | BDSC | BDSC:35144; RRID:BDSC_35144 | RNAi of *Tsc1* |
| Genetic reagent (*D. melanogaster*) | *Tsc1^Ri^* | BDSC | BDSC:31039; RRID:BDSC_31039 | RNAi of *Tsc1* (strong) |
| Genetic reagent (*D. melanogaster*) | *Obp56e* | VDRC | VDRC:3355 | RNAi of *Obp56e* |
| Genetic reagent (*D. melanogaster*) | *Mco3* | VDRC | VDRC:43288 | RNAi of *Mco3* |
| Genetic reagent (*D. melanogaster*) | *mah* | VDRC | VDRC:1571 | RNAi of *mah* |
| Genetic reagent (*D. melanogaster*) | *mah* | BDSC | BDSC:53701; RRID:BDSC_53701 | RNAi of *mah* |
| Genetic reagent (*D. melanogaster*) | *CG9896* | VDRC | VDRC ID# 107926 | RNAi of *CG9896* |
| Genetic reagent (*D. melanogaster*) | *CG9896* | BDSC | BDSC:42587; RRID:BDSC_42587 | RNAi of *CG9896* |
| Genetic reagent (*D. melanogaster*) | *CG15282* | VDRC | VDRC:7712 | RNAi of *CG15282* |
| Genetic reagent (*D. melanogaster*) | *CG42749* | VDRC | VDRC:28574 | RNAi of *CG42749* |
| Genetic reagent (*D. melanogaster*) | *CG42749* | VDRC | VDRC:51132 | RNAi of *CG42749* |
| Genetic reagent (*D. melanogaster*) | *CG42749* | VDRC | VDRC:51133 | RNAi of *CG42749* |
| Genetic reagent (*D. melanogaster*) | *Hsp22/CG4456* | VDRC | VDRC:43632 | RNAi of *Hsp22/CG4456* |
| Genetic reagent (*D. melanogaster*) | *Hsp22/CG4456* | BDSC | BDSC:41709; RRID:BDSC_41709 | RNAi of *Hsp22/CG4456* |
| Genetic reagent (*D. melanogaster*) | *Hsp22/CG4456* | BDSC | BDSC:51397;  RRID:BDSC_51397 | RNAi of *Hsp22/CG4456* |
| Genetic reagent (*D. melanogaster*) | *Hsp22/CG4456* | VDRC | VDRC:49795 | RNAi of *Hsp22/CG4456* |
| Genetic reagent (*D. melanogaster*) | *Hsp22/CG4456* | VDRC | VDRC:49796 | RNAi of *Hsp22/CG4456* |
| Genetic reagent (*D. melanogaster*) | *clos* | VDRC | VDRC:104142 | RNAi of *clos* |
| Genetic reagent (*D. melanogaster*) | *clos* | VDRC | VDRC:108711 | RNAi of *clos* |
| Genetic reagent (*D. melanogaster*) | *clos* | BDSC | BDSC:52966; RRID:BDSC_52966 | RNAi of *clos* |
| Genetic reagent (*D. melanogaster*) | *CG40472* | VDRC | VDRC:109239 | RNAi of *CG40472* |
| Genetic reagent (*D. melanogaster*) | *CG10424* | VDRC | VDRC:39667 | RNAi of *CG10424* |
| Genetic reagent (*D. melanogaster*) | *Fili* | VDRC | VDRC:106055 | RNAi of *Fili* |
| Genetic reagent (*D. melanogaster*) | *Fili* | BDSC | BDSC:28568; RRID:BDSC_28568 | RNAi of *Fili* |
| Genetic reagent (*D. melanogaster*) | RNAi of *CG43313* | VDRC | VDRC:106610 | RNAi of *CG43313* |
| Genetic reagent (*D. melanogaster*) | *CG43313* | BDSC | BDSC:53990; RRID:BDSC_53990 | RNAi of *CG43313* |
| Genetic reagent (*D. melanogaster*) | *CG6766* | VDRC | VDRC:38035 | RNAi of *CG6766* |
| Genetic reagent (*D. melanogaster*) | *CG6766* | BDSC | BDSC:42924; RRID:BDSC_42924 | RNAi of *CG6766* |
| Genetic reagent (*D. melanogaster*) | *CG6766* | BDSC | BDSC:55745; RRID:BDSC_55745 | RNAi of *CG6766* |
| Genetic reagent (*D. melanogaster*) | *CG2865* | BDSC | BDSC:43165; RRID:BDSC_43165 | RNAi of *CG2865* |
| Genetic reagent (*D. melanogaster*) | *Ant2* | VDRC | VDRC:102533 | RNAi of *Ant2* |
| Genetic reagent (*D. melanogaster*) | *Ptp4E* | VDRC | VDRC:1012 | RNAi of *Ptp4E* |
| Genetic reagent (*D. melanogaster*) | *Ptp4E* | VDRC | VDRC:1013 | RNAi of *Ptp4E* |
| Genetic reagent (*D. melanogaster*) | *Ptp4E* | VDRC | VDRC:4297 | RNAi of *Ptp4E* |
| Genetic reagent (*D. melanogaster*) | *Ptp4E* | VDRC | VDRC:27232 | RNAi of *Ptp4E* |
| Genetic reagent (*D. melanogaster*) | *Ptp4E* | BDSC | BDSC:38369; RRID:BDSC_38369 | RNAi of *Ptp4E* |
| Genetic reagent (*D. melanogaster*) | *Ptp4E* | BDSC | BDSC:60008; RRID:BDSC_60008 | RNAi of *Ptp4E* |
| Genetic reagent (*D. melanogaster*) | *Hexo1* | VDRC | VDRC:102247 | RNAi of *Hexo1* |
| Genetic reagent (*D. melanogaster*) | *Hexo1* | BDSC | BDSC:67312; RRID:BDSC_67312 | RNAi of *Hexo1* |
| Genetic reagent (*D. melanogaster*) | *grnd* | VDRC | VDRC:43454 | RNAi of *grnd* |
| Genetic reagent (*D. melanogaster*) | *grnd* | VDRC | VDRC:104538 | RNAi of *grnd* |
| Genetic reagent (*D. melanogaster*) | *Rpt5* | VDRC | VDRC:105133 | RNAi of *Rpt5* |
| Genetic reagent (*D. melanogaster*) | *Rpt5* | BDSC | BDSC:32422; RRID:BDSC_32422 | RNAi of *Rpt5* |
| Genetic reagent (*D. melanogaster*) | *Rpt5* | BDSC | BDSC:53886; RRID:BDSC_53886 | RNAi of *Rpt5* |
| Genetic reagent (*D. melanogaster*) | *CG8860* | VDRC | VDRC:102745 | RNAi of *CG8860* |
| Genetic reagent (*D. melanogaster*) | *CG8860* | BDSC | BDSC:60127; RRID:BDSC_60127 | RNAi of *CG8860* |
| Genetic reagent (*D. melanogaster*) | *Edem1* | VDRC | VDRC:6923 | RNAi of *Edem1* |
| Genetic reagent (*D. melanogaster*) | *α-Man-Ia* | VDRC | VDRC:100654 | RNAi of *α-Man-Ia* |
| Genetic reagent (*D. melanogaster*) | *α-Man-Ib* | VDRC | VDRC:4419 | RNAi of *α-Man-Ib* |
| Genetic reagent (*D. melanogaster*) | *Pdi* | VDRC | VDRC:23359 | RNAi of *Pdi* |
| Genetic reagent (*D. melanogaster*) | *prtp* | VDRC | VDRC:106924 | RNAi of *prtp* |
| Genetic reagent (*D. melanogaster*) | *Ssrβ* | VDRC | VDRC:12101 | RNAi of *Ssrβ* |
| Genetic reagent (*D. melanogaster*) | *Sec61α* | VDRC | VDRC:109660 | RNAi of *Sec61α* |
| Genetic reagent (*D. melanogaster*) | *Sec61β* | VDRC | VDRC:107784 | RNAi of *Sec61β* |
| Genetic reagent (*D. melanogaster*) | *Sec61γ* | VDRC | VDRC:100603 | RNAi of *Sec61γ* |
| Genetic reagent (*D. melanogaster*) | *CG13426* | VDRC | VDRC:107528 | RNAi of *CG13426* |
| Genetic reagent (*D. melanogaster*) | *TRAM* | VDRC | VDRC:39187 | RNAi of *TRAM* |
| Genetic reagent (*D. melanogaster*) | *Der-1* | VDRC | VDRC:44210 | RNAi of *Der-1* |
| Genetic reagent (*D. melanogaster*) | *Der-1* | VDRC | VDRC:44211 | RNAi of *Der-1* |
| Genetic reagent (*D. melanogaster*) | *Der-2* | VDRC | VDRC:108440 | RNAi of *Der-2* |
| Genetic reagent (*D. melanogaster*) | *sip3* | BDSC | BDSC:50609; RRID:BDSC_50609 | RNAi of *sip3* |
| Genetic reagent (*D. melanogaster*) | *sip3* | BDSC | BDSC:61344; RRID:BDSC_61344 | RNAi of *sip3* |
| Genetic reagent (*D. melanogaster*) | *Hrd3* | BDSC | BDSC:62368; RRID:BDSC_62368 | RNAi of *Hrd3* |
| Genetic reagent (*D. melanogaster*) | *CG40045* | VDRC | VDRC:109167 | RNAi of *CG40045* |
| Genetic reagent (*D. melanogaster*) | *PEK* | BDSC | BDSC:35162; RRID:BDSC_35162 | RNAi of *PEK* |
| Genetic reagent (*D. melanogaster*) | *PEK* | BDSC | BDSC:42499; RRID:BDSC_42499 | RNAi of *PEK* |
| Genetic reagent (*D. melanogaster*) | *Gcn2* | VDRC | VDRC:32664 | RNAi of *Gcn2* |
| Genetic reagent (*D. melanogaster*) | *eIF2α* | VDRC | VDRC:104562 | RNAi of *eIF2α* |
| Genetic reagent (*D. melanogaster*) | *Atf6* | VDRC | VDRC:36504 | RNAi of *Atf6* |
| Genetic reagent (*D. melanogaster*) | *wfs1* | VDRC | VDRC:108932 | RNAi of *wfs1* |
| Genetic reagent (*D. melanogaster*) | *Ire1* | BDSC | BDSC:36743; RRID:BDSC_36743 | RNAi of *Ire1* |
| Genetic reagent (*D. melanogaster*) | *Ire1* | BDSC | BDSC:62156; RRID:BDSC_62156 | RNAi of *Ire1* |
| Genetic reagent (*D. melanogaster*) | *Ire1* | BDSC | BDSC:35253; RRID:BDSC_35253 | RNAi of *Ire1* |
| Genetic reagent (*D. melanogaster*) | *Cul1* | VDRC | VDRC:108558 | RNAi of *Cul1* |
| Genetic reagent (*D. melanogaster*) | *SkpA* | VDRC | VDRC:107815 | RNAi of *SkpA* |
| Genetic reagent (*D. melanogaster*) | *STUB1* | VDRC | VDRC:107447 | RNAi of *STUB1* |
| Genetic reagent (*D. melanogaster*) | *p47* | VDRC | VDRC:107148 | RNAi of *p47* |
| Genetic reagent (*D. melanogaster*) | *Csp* | VDRC | VDRC:34168 | RNAi of *Csp* |
| Genetic reagent (*D. melanogaster*) | *TER94* | VDRC | VDRC:24354 | RNAi of *TER94* |
| Genetic reagent (*D. melanogaster*) | *Ufd1*-*like* | VDRC | VDRC:24700 | RNAi of *Ufd1*-*like* |
| Genetic reagent (*D. melanogaster*) | *CG4603* | VDRC | VDRC:21894 | RNAi of *CG4603* |
| Genetic reagent (*D. melanogaster*) | *Plap* | VDRC | VDRC:110732 | RNAi of *Plap* |
| Genetic reagent (*D. melanogaster*) | *Ubqn* | VDRC | VDRC:106050 | RNAi of *Ubqn* |
| Genetic reagent (*D. melanogaster*) | *Ubqn* | VDRC | VDRC:47447 | RNAi of *Ubqn* |
| Genetic reagent (*D. melanogaster*) | *Ubqn* | VDRC | VDRC:47448 | RNAi of *Ubqn* |
| Genetic reagent (*D. melanogaster*) | *CG10694* | VDRC | VDRC:100212 | RNAi of *CG10694* |
| Genetic reagent (*D. melanogaster*) | *Rad23* | VDRC | VDRC:104354 | RNAi of *Rad23* |
| Genetic reagent (*D. melanogaster*) | *CG6766^140290^* | Kyoto Drosophila Genetic Resource Center | DGGR:140290, RRID:DGGR_140290 |  |
| Genetic reagent (*D. melanogaster*) | *UAS-Xbp1-EGFP* | BDSC | BDSC:60730; RRID:BDSC_60730 |  |
| Cell line (*D. melanogaster*) | Cell line: Kc167 | Drosophila Genomics Resource Center | DGRC:1, RRID:CVCL_Z834 |  |
| Antibody | Rabbit anti-FoxO | Dr. Mark Tatar | PMID: 22935001 |  |
| Antibody | Mouse anti-Cnx99A | Developmental Studies Hybridoma Bank | Cat# Cnx99A 6-2-1, RRID:AB_2722011 |  |
| Antibody | Mouse anti-α-tubulin | Sigma-Aldrich | Cat#T9026, RRID:AB_477593 |  |
| Antibody | Mouse anti-GFP | Thermo Fisher Scientific | Cat#A-11120, RRID:AB_221568 |  |
| Chemical compound, drug | DMSO | Carl Roth | Cat#A994.1 |  |
| Chemical compound, drug | Tunicamycin | Sigma-Aldrich | Cat#T7765 |  |
| Chemical compound, drug | Thapsigargin | Sigma-Aldrich | Cat#T9033 |  |
| Chemical compound, drug | MG132 | Sigma-Aldrich | Cat#C2211 |  |
| Chemical compound, drug | Eeyarestatin I | Sigma-Aldrich | Cat#E1286 |  |
| Commercial assay, kit | Ovation SoLo RNA-Seq System Drosophila | NuGEN | Cat#0502-32 |  |
| Software, algorithm | STAR | PMID:  23104886 | https://github.com/alexdobin/STAR |  |
| Software, algorithm | featureCounts | PMID:  24227677 | https://bio.tools/featurecounts |  |
| Software, algorithm | edgeR | PMID:  19910308 | https://doi.org/doi:10.18129/B9.bioc.edgeR |  |
| Software, algorithm | DAVID | PMID:  19131956 | https://david.ncifcrf.gov |  |
| Software, algorithm | GSEA | PMID:  16199517 | http://software.broadinstitute.org/gsea/index.jsp |  |
| Software, algorithm | ImageJ | PMID:  22743772 | https://imagej.net/Fiji |  |
| Software, algorithm | R Studio | r-project.org | https://www.rstudio.com |  |
| Software, algorithm | Adobe Illustrator | Adobe | CC 2017 |  |
