# Supplementary material for: FoxO suppresses endoplasmic reticulum stress to inhibit growth of Tsc1-deficient tissues under nutrient restriction
Source: eLife. 2020 Jun 11;9:e53159. doi: 10.7554/eLife.53159 (PMC7289595; doi:10.7554/eLife.53159)
Supplement: Supplementary file 2. [file elife-53159-supp2.docx]

**Primer sequences for dsRNA synthesis and qPCR**

| **Primers for dsRNA synthesis** | |
| --- | --- |
| **Name** | **Sequence** |
| EGFP forward | CGCTAATACGACTCACTATAGGGAGATCACCGGGGTGGTGCCCATCCTGG |
| EGFP reverse | CGCTAATACGACTCACTATAGGGAGATGCCGAGAGTGATCCCGGCGGCGG |
| foxo forward | CGCTAATACGACTCACTATAGGGAGACACAACCGCTTTATGAGGGT |
| foxo reverse | CGCTAATACGACTCACTATAGGGAGACTCTCGGGAAAGTGATCCAG |
| **Primers for qPCR** | |
| **Name** | **Sequence** |
| foxo forward | AGGCGCAGCCGATAGACGAATTTA |
| foxo reverse | TGCTGTTGACCAGGTTCGTGTTGA |
| Hsc-70-3 forward | GAATCAGTTGACCACCAATCCC |
| Hsc-70-3 reverse | AACTTGATGTCGTGTTGCACA |
| PEK forward | CTGCGCAGTCTTCGGGACGG |
| PEK reverse | AGCTGCTGAAGGTGCGGCTG |
| spliced-Xbp1 forward | CAACCTTGGATCTGCCGCAGGG |
| spliced-Xbp1 reverse | CGCTCCAGCGCCTGTTTCCAG |
| RpS23 forward | TCGTAACGCTCAGCAACG |
| RpS23 reverse | GCAGACCTCTTGGCTTGC |
